# Structural Characterization of Polymers by MALDI Spiral-TOF Mass Spectrometry Combined with Kendrick Mass Defect Analysis

**DOI:** 10.1007/s13361-014-0915-y

**Published:** 2014-05-21

**Authors:** Hiroaki Sato, Sayaka Nakamura, Kanae Teramoto, Takafumi Sato

**Affiliations:** 1 Research Institute for Environmental Management Technology, National Institute of Advanced Industrial Science and Technology (AIST), Tsukuba, Japan; 2 Advanced Technology Department, JEOL Ltd, Akishima, Japan; 3 MS Business Unit, JEOL Ltd, Akishima, Japan

**Keywords:** Kendrick mass defect analysis, MALDI spiral-TOFMS, High-resolution mass spectrometry, Polymer characterization

## Abstract

High-resolution mass spectrometry (HRMS) continues to play an important role in the compositional characterization of larger organic molecules. In the field of polymer characterization, however, the application of HRMS has made only slow progress because of lower compatibility between matrix-assisted laser desorption/ionization (MALDI) and ultrahigh-resolution Fourier transform ion cyclotron resonance mass spectrometry (FT-ICRMS). In this study, a newly developed type of MALDI high-resolution time-of-flight mass spectrometry (TOFMS) with a spiral ion trajectory (MALDI spiral-TOFMS) was applied to the structural and compositional characterization of polymers. To create a graphical distribution of polymer components on a two-dimensional plot converted from complex mass spectra, we adopted a slightly modified Kendrick mass defect (KMD) analysis based on accurate masses determined using spiral-TOFMS. By setting the Kendrick mass scale based on the mass of the repeating units of a given polymer, components with common repeat units lined up in the horizontal direction on the KMD plot, whereas those components with different structures were shifted vertically. This combination of MALDI spiral-TOFMS measurement and KMD analysis enabled the successful discrimination of the polymer components in a blend of poly(alkylene oxide)s, the compositional analysis of poly(ethylene oxide)/poly(propylene oxide) block copolymers, and profiling of the end-group distribution of poly(ε-caprolactone)s synthesized under different conditions.

**Figure:**
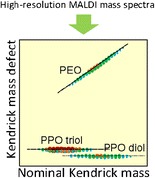
ᅟ

ᅟ

## Introduction

Polymer characterization has the potential to be particularly useful in assisting the design of sophisticated polymeric materials with dedicated functions. Matrix-assisted laser desorption/ionization time-of-flight mass spectrometry (MALDI-TOFMS) is an important tool in this technique [1, 2]. Currently, conventional TOFMS instruments used for polymer characterization apply a single reflector with a flight path of approximately 2 m. A recently-developed high-resolution MALDI-TOFMS with a spiral ion trajectory, termed “MALDI spiral-TOFMS” [3, 4], has a long flight path of approximately 17 m (2.1 m × 8 turns) along the spiral ion trajectory, which yields a high mass-resolving power of 80,000 at full width at half maximum (FWHM) for *m/z* ca. 2500 of a peptide sample. In a previous study, we applied MALDI spiral-TOFMS to the detailed structural characterization of polyphenols [5, 6] and radical-polymerized copolymers [7]. In these former studies [5, 6], we were able to identify the structures of repeating units of polyphenols. In the latter study [7], copolymer compositions and several types of end-group combinations could be identified, allowing the compositional distribution to be evaluated. In both cases, MALDI spiral-TOFMS could be used to provide an accurate judgment of the mass differences at ca. 16 Da to discriminate O and CH_4_, between which the mass difference is only 0.036 Da. We therefore anticipate MALDI spiral-TOFMS to make a significant contribution to the development of the structural characterization of polymers.

Since analysis of complicated samples by high-resolution mass spectrometry necessarily deals with an enormous collection of peak data, an effective data processing method, preferably one that does not rely on peak assignments, is required. Kendrick mass defect (KMD) analysis is a potentially effective method of identifying homologous series differing only by a number of base units [8–10]. The Kendrick mass is a new mass scale designed to supersede the IUPAC mass scale. Usually the Kendrick mass of CH_2_ is defined as exactly 14, but the Kendrick mass of a given compound can be obtained by multiplication of the mass values by 14/14.01565, where 14.01565 is the IUPAC mass of CH_2_. However, the Kendrick mass is not limited to the CH_2_ base: other groups can also be used to define the Kendrick mass to suit the task in hand. KMD is defined as the difference between the exact Kendrick mass and the nominal Kendrick mass (NKM). Two-dimensional plots of KMD as a function of NKM display the distribution of components, in which the components with common repeat units line up in the horizontal direction, whereas the components having different structures shift in the vertical direction.

KMD analysis has been used chiefly to characterize petroleum [9, 11], natural organic matter [12, 13], and lipid samples [14, 15] by means of ultrahigh-resolution Fourier transform ion cyclotron resonance MS (FTICR-MS) combined with electrospray ionization (ESI). FTICR-MS can separate isobaric peaks at a resolution over 100 k. However, the observation of molecular weight distribution of polymers using a combination of MALDI and FTICR-MS encounters problems, mainly caused by mass discrimination when ions are trapped and stored in the ICR cell [16–19]. Although the superb resolving power and mass accuracy of FTICR-MS make it an eminently useful technique, MALDI-FTICR-MS is not ideal for polymer analysis at this stage. In fact, only a few polymer characterizations by MALDI-FTICR-MS have been described [20–23], and, to our knowledge, no use of KMD analysis for polymer characterization has yet been reported.

Because MALDI spiral-TOFMS should be sufficient to determine each peak with a high-mass accuracy of within a few ppm, even for isobaric peaks, in this study we applied MALDI spiral-TOFMS combined with KMD analysis to polymer characterization. Since KMD analysis is powerful means to differentiate similar mixtures, this study has demonstrated structural distribution analyses, which include discrimination of the polymer components in the blend, compositional analysis of copolymers, and the profiling of end-group distribution of polymers synthesized under different conditions.

## Experimental

### Polymer Samples

Several types of poly(alkylene oxide) and poly(ε-caprolactone) (PCL) were used as model samples. The chemical structures, molecular weights, and suppliers are summarized in Table 1. Diol and triol types of poly(propylene oxide) (PPO) were purchased from Wako Pure Chemical Industries (Osaka, Japan). Poly(ethylene oxide) (PEO) and triblock copolymer, PEO-*block*-PPO-*block*-PEO [P(EO-*b*-PO)], were purchased from Sigma-Aldrich Japan (Tokyo, Japan). Four types of PCL synthesized under different conditions (PCL-1–4) were purchased from Sigma-Aldrich or Polymer Source (Montreal, Canada). All samples were used as received. The methanol solutions of PEO, PPO-diol, and PPO-triol (ca. 1 mg/mL) were prepared and mixed with 1/1/1 (v/v/v) to make a blend sample. P(EO-*b*-PO) was dissolved in methanol (at ca. 1 mg/mL). Each PCL sample was dissolved in tetrahydrofuran (THF) at a concentration of ca. 1 mg/mL.

**Table 1:** The Names of the Polymer Samples Used in this Study Together with Probable Chemical Structures, Average Molecular Weight, and Suppliers

Sample name	Probable chemical structure^a^	Molecular weight	Supplier and product code
Poly(alkylene oxide) samples			
PEO	HO-(EO)_n_-H	*M*_n_ = 2050	Sigma-Aldrich, 295906-5G
PPO-diol	HO-(PO)_n_-H	ca. 2000	Wako, 164-05895
PPO-triol		ca. 1500	Wako, 164-17625
P(EO-*b*-PO)	HO-(EO)_a_-(PO)_b_-(EO)_c_-H (a + c)/b = 50/50 (w/w) = 44/56 (mol/mol)	ca. 1900	Sigma-Aldrich, 435414-250ML
Poly(ε-caprolactone) samples			
PCL-1	HO-(CL)_x_-(CH_2_CH_2_O)_2_-(CL)_y_-H	ca. 10000	Sigma-Aldrich, 440752-250G
PCL-2	(CH_3_)_2_CHO-(CL)_n_-H	*M*_n_ = 8000 *M*_w_ = 10000	Polymer Source, P1302-CL
PCL-3	(CH_3_)_2_CHO-(CL)_n_-H	*M*_n_ = 7700 *M*_w_ = 8900	Polymer Source, P1933-CPL
PCL-4	C_2_H_5_O-(CL)_n_-H	*M*_n_ = 3500 *M*_w_ = 5200	Polymer Source, P1934-CPL

^a^EO=CH_2_CH_2_O, PO=CH(CH_3_)CH_2_O, CL=CO(CH_2_)_5_O

### MALDI Spiral-TOFMS Measurement

As the matrix for sample ionization, 2,5-dihydroxybenzoic acid (DHB) purchased from Wako was employed. About 10 mg of DHB was dissolved in methanol for the poly(alkylene oxide) samples or THF for the PCL samples. Next, about 1 μL of the sample/matrix (1/10 v/v) mixture was pipetted onto the stainless steel target plate, which was then dried in air. MALDI mass spectra were observed using a JEOL JMS-S3000 Spiral-TOFMS (JEOL, Tokyo, Japan). The details of the instrument’s configuration are described in reference [3]. Ions generated by irradiation with a 349-nm Nd:YLF laser were accelerated at 20 kV. The ions then passed along a spiral ion trajectory with a flight length of approximately 17 m. The settings of delay time and grid voltage were optimized to maintain ∆M < ca. 0.03 Da at FWHM over the range of *m/z* 800–3000. Mass calibration was made using a poly(methyl methacrylate) (PMMA) standard (peak-top molecular weight, *M*_p_ = 1310) purchased from Polymer Laboratories (Church Stretton, UK).

### Data Processing Procedure on Kendrick Mass Defect Analysis

In the Kendrick mass defect analysis, at the beginning the observed accurate mass values on the IUPAC mass scale are converted to the Kendrick mass (KM) according to the following equation:

1 $$ KM= observed\  IUPAC\  mass\times \frac{ nominal\  mass\  of\  base\  unit}{ IUPAC\  mass\  of\  base\  unit} $$

In many cases, the methylene unit is set as the base unit (i.e., CH_2_ = 14.01565 Da is converted to 14) [8, 9]. In polymer analyses, however, the Kendrick mass scale based on the mass of the repeating units of a given polymer would be useful for easily depicting the distribution of homologous series. The KM values are composed of two parts (i.e., nominal Kendrick mass (NKM) and Kendrick mass defect (KMD). The NKM is the nearest integer of KM, whereas the KMD is the difference between NKM and KM.

2 $$ KMD= NKM- KM $$

The Kendrick plot is the two-dimensional graph with NKM on the x-axis and KMD on the y-axis. In this plot, homologous series having a common base unit should line up in the horizontal direction.

## Results and Discussion

### Distribution of Polymer Components in the Blend

Poly(alkylene oxide)s such as PEO, PPO, and their modified polymers are widely used in industrial, agricultural, and domestic applications as moisturizing agents, emulsifiers, surfactants, and so on. In many cases, several kinds of poly(alkylene oxide)s are blended to achieve the desired conditions. Understanding the blend conditions is important from a quality control perspective.

**Figure 1:**
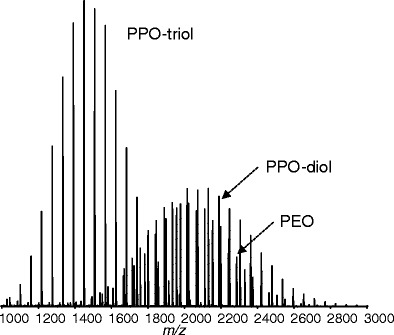
MALDI mass spectrum of the blend of PEO, PPO-diol, and PPO-triol samples with a ratio of 1:1:1 (w/w/w)

Figure 1 shows the mass spectrum of the blend of PEO, PPO-diol, and PPO-triol with a ratio of 1:1:1 (w/w/w). A bimodal peak distribution can be observed with maxima at *m/z* ca. 1500 and *m/z* ca. 2000. The former distribution corresponds to PPO-triol, with an average molecular weight of ca. 1500. The latter distributions are likely to overlap with the peaks of the PEO and PPO-diol samples. The obtained mass resolution values were 46100 at *m/z* 1450 and 71500 at *m/z* 2130, achieving almost constant ∆M at FWHM of ca. 0.03 Da over the observed mass range. Mass accuracy fell within the range of 2 ppm. Resolving power and mass accuracy of this degree is likely to be sufficient to perform KMD analysis.

**Figure 2:**
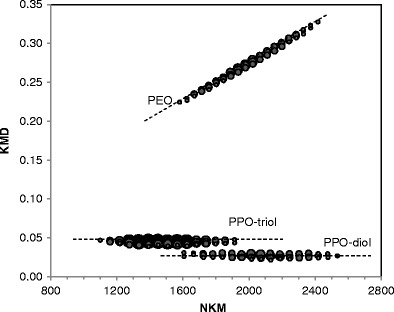
Two-dimensional plots of KMD versus NKM of the blend sample using a mass scale based on PO units. The size of each dot indicates peak intensity. The peaks with more than 5% relative intensities were plotted

The values of NKM and KMD were calculated as described in the Experimental section. In this case, we chose PO units as the base unit in Equation 1 (C_3_H_6_O = 58.04187 Da was converted to 58). Figure 2 shows two-dimensional plots of KMD versus NKM of the blend sample. Here, the relative intensities (5%–100%) of the observed peaks are scaled by dot diameter and concentration. The KMD plot shows that two types of PPO chains are separately distributed in the horizontal direction, whereas the PEO chains line up obliquely. The swelling of the distribution lines is caused by isotope distribution. It is noteworthy that the overlapping distribution of PEO and PPO-diol between *m/z* 1500 and 2500 in Figure 1 can be clearly separated on the KMD plot.

The theoretical KMD value of PPO-diol ions ([HO-(C_3_H_6_O)_n_-H+Na]^+^) is obtained as 0.029, because the residual mass of PPO-diol is 41.000 Da (H_2_ONa) and its KM and NKM can be obtained as 40.971 and 41. In the same manner, the theoretical KMD values of PPO-triol ions can be calculated as 0.046, attributed from the residual composition of C_3_H_8_O_3_Na. Thus, the two horizontal lines in Figure 1 are specific to each PPO homologue. As for PEO chains, the atomic composition of the EO unit is C_2_H_4_O, which corresponds to 44.026 Da or KM = 43.994. In other words, the KMD value of PEO is increased by 0.006 on increasing NKM = 44 for one EO unit, with the result that the slope of the lines of PEO homologues is theoretically the same, without depending on residual structures such as end-groups. The residual structures influence the intercept value of the distribution lines. Thus, the slope and intercept values of the distribution lines on the KMD plot are specific to the atomic composition of polymers. KMD analysis is thus useful not only for discriminating component polymers but also for rapidly identifying the types of components if the reference values are prepared beforehand.

### Distribution of Copolymer Compositions

In general, block copolymers of poly(alkylene oxide)s show surfactant properties that can be used in various industrial applications, cosmetics, pharmaceuticals, and so on. The chemical structures of copolymers, such as copolymer composition, distribution, and block length, are likely to have a strong influence on surfactant properties. In this study, KMD analysis was applied to the structural characterization of the P(EO-*b*-PO) sample as an ABA-type triblock copolymer.

**Figure 3:**
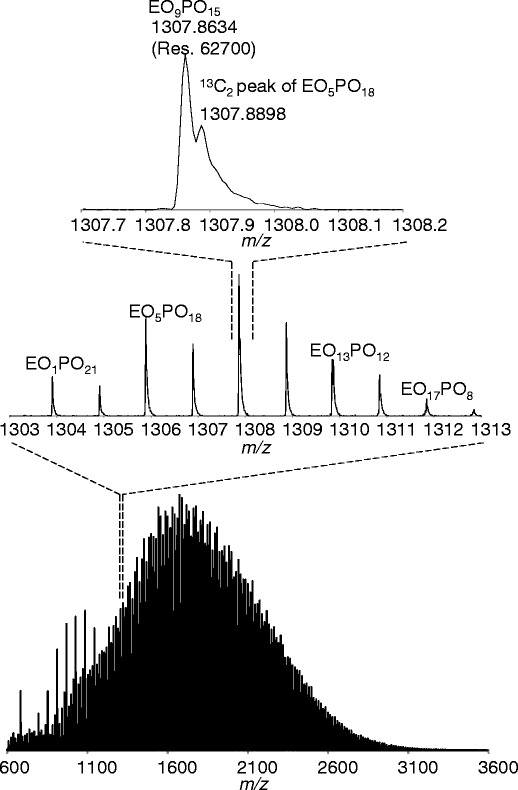
Mass spectra of P(EO-b-PO). Broad-band mass spectrum (bottom); expanded mass spectrum in the range *m/z* 1303–1313 (middle); *m/z* 1307.7–1308.2 (top)

Figure 3 shows the mass spectra of P(EO-*b*-PO), illustrating the broad-band mass spectrum and the expanded mass spectra. The peaks are chiefly distributed in the range *m/z* 800–3000 with the maxima at *m/z* ca. 1800. The mass spectra of P(EO-*b*-PO) are composed of peaks with a 2-Da interval that corresponds to the mass differences between EO_x_PO_y_ and EO_x+4_PO_y-3_, as shown in the expanded spectra in Figure 3. Each peak further overlaps the second isotope peak. For example, close to the monoisotope peak of EO_9_PO_15_ at *m/z* 1307.8634, the shoulder peaks can be seen at *m/z* 1307.8898, which is the second isotope peak of EO_5_PO_18_. These mass differences in ca. 0.027 Da can be resolved by using spiral-TOFMS.

**Figure 4:**
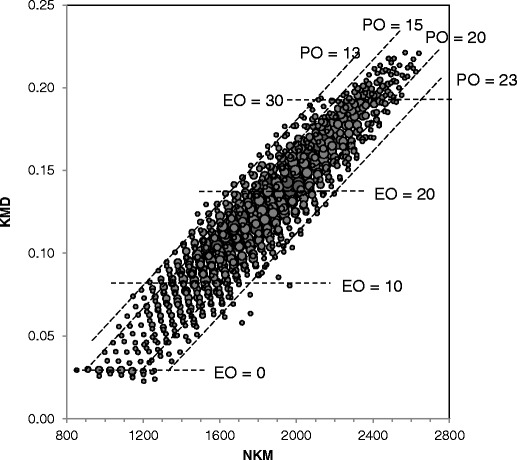
KMD plot of P(EO-b-PO). The lines indicate the theoretical distribution of homologues with the same numbers of EO or PO compositions. The size of each dot indicates peak intensity. The peaks with more than 10% relative intensities were plotted

Figure 4 shows the KMD plot based on the PO unit of the P(EO-*b*-PO) sample. All of the observed peaks, including isotope peaks, with more than 10% relative intensities were collectively converted to a set of NKM-KMD values and plotted without any peak-picking or de-isotoping procedures. The components are clearly dispersed upward to the right, reflecting their EO/PO compositional distribution. The copolymer chains with the same numbers of EO units but different numbers of PO units line up in a horizontal distribution at intervals of 58. As for the copolymer chains with the same numbers of PO units, NKM increases by 44 and KMD value increases by 0.0055. The distribution of the components tended to range between EO = 0–35 and PO = 13–23, as indicated by the dotted lines in Figure 4. It should be noted that the string of dots on the line of EO = 0 indicates the presence of the homopolymer of PO. Thus, the resulting KMD plot suggests that the PEO block would be elongated from both ends of the core PPO block, of which the degree of polymerization was distributed from *n* = 13–23. The centroid of the dot distribution was obtained as NKM = ca. 1870 and KMD = ca. 0.140. Here, the NKM and KMD vales of P(EO-*b*-PO) can be calculated according to the following equations,

3 $$ NKM={n}_{EO}\times 44+{n}_{PO}\times 58+18+23 $$

4 $$ KMD=0.0055\operatorname{l}{n}_{PO}+0.0293 $$

where ${n}_{EO}$ and ${n}_{PO}$ are the numbers of the EO and PO units, and the integral numbers (44, 58, 18, and 23) are the NKM values of the EO and PO units, end-groups, and sodium cation. In Equation 4, 0.0055 is the KMD value of the EO unit and 0.0293 is that of the sum of end-groups and sodium cation (H_2_ONa). Thus, ${n}_{EO}$ and ${n}_{PO}$ can be calculated using the equations derived from Equations 3 and 4 as follows:

5 $$ {n}_{PO}=\left( KMD-0.0293\right)/0.0055 $$

6 $$ {n}_{EO}=\left( NKM-{n}_{PO}\times 58-41\right)/44 $$

According to Equations 5 and 6, the average copolymer composition of this sample can be obtained as EO_15.0_PO_20.2_. This value corresponds to an EO composition of 42.6 mol%, which is in good agreement with the value of ca. 44 mol% provided by the supplier. As demonstrated above, the KMD plot can accurately depict the compositional distribution of copolymers.

### Profiling of End-Group Distribution

End-groups (including initiators incorporated into polymer chains) contain detailed information about the synthetic process, degradation profiles, or chemical modifications applied to enhance the functionality of polymers. End-group determination using MALDI-MS is thus a key element in polymer characterization. The mass of end-groups (*M*_end_) can be given by

7 $$ {M}_{end}={M}_{obs}-{M}_{monomer}\times n-{M}_{cation} $$

where *M*_obs_, *M*_monomer_, and *M*_cation_ are the masses of the observed ion, monomer unit, and cation, respectively, and *n* is the degree of polymerization. Even if the sample is unknown, *M*_monomer_ can be determined from the regular peak interval and *M*_cation_ can be known from the used cationization reagent. Here, a difficulty arises in the determination of *n*. In many cases, *n* is tentatively estimated on the assumption of *M*_end_ < *M*_monomer_. However, one should allow for the possibility that *M*_end_ > *M*_monomer_. In addition, end-group combinations in a given polymer are not always uniform, and it would be even more complicated to determine the various *M*_end_ values of each end-group combination. To solve this problem, we propose a modified KMD analysis to depict the distribution of end-group combinations. In this paper, we demonstrate the profiling of end-group distribution of PCL samples synthesized using different procedures.

**Figure 5:**
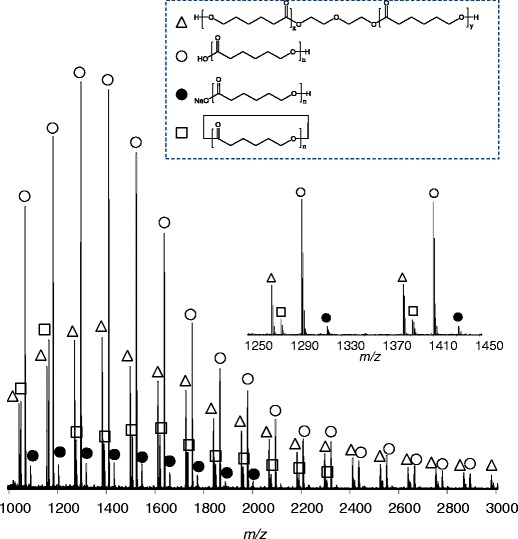
Mass spectrum of PCL-1 sample

Figure 5 shows the MALDI mass spectra of the PCL-1 sample. This polymer is synthesized by ring-opening polymerization of ε-caprolactone (CL) with diethylene glycol as an initiator. As a result, the main components of the PCL-1 sample contain a diethylene glycol unit and two hydroxyl terminals (PCL-diol). In addition, cyclic PCL (cyc-PCL) and linear PCL chains end-capped with carbonic acid and hydroxyl groups (carboxyl-PCL) are generated as by-products [24]. The occurrence of sodium salts of carboxyl-PCL is an artifact of MALDI-MS.

**Figure 6:**
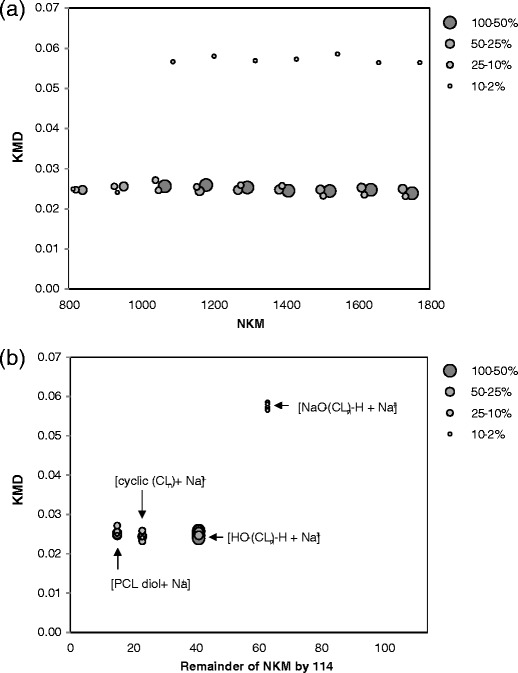
KMD plot of PCL-1 sample **(a)** and RKM-KMD plot **(b)**. The size of each dot indicates peak intensity. The peaks with more than 2% relative intensities were plotted

The mass spectral data with more than 2% relative intensities were converted to make the KMD plot shown in Figure 6a, in which the 114.06808 Da (C_6_H_10_O_3_) of the CL unit is converted to 114. The dots on the plot are chiefly distributed around the horizontal line of KMD = ca. 0.024. These series have overlapping data for PCL-diol, cyc-PCL, and carboxyl-PCL because the theoretical KMD values of these components are close to 0.024. The minor series at KMD = ca. 0.06 corresponds to the sodium salt of carboxyl-PCL. When several polymer chains with different end-group combinations have very close KMD values, it is difficult to discriminate them on the KMD plot. In a different approach, we have proposed that the remainder of NKM (RKM) divided by the nominal Kendrick mass of the repeating unit (in this case 114) was used as the x-axis in the modified KMD plot. NKM of the observed peaks (*NKM*_obs_) can be given by

8 $$ NK{M}_{obs}= NK{M}_{monomer}\times n+ NK{M}_{end}+ NK{M}_{cation} $$

where *NKM*_monomer_, *NKM*_end_, and *NKM*_cation_ are the NKM of monomer unit, end-groups, and cation, respectively. If the both sides of Equation 8 are divided by *M*_monomer_, we can obtain

9 $$ \frac{ NK{M}_{obs}}{ NK{M}_{monomer}}=n+\frac{ NK{M}_{end}+ NK{M}_{cation}}{ NK{M}_{monomer}}=\left(n+a\right)+ RKM\ \mathrm{and}\ 0\le RKM< NK{M}_{monomer} $$

where, (*n* + *a*) and *RKM* correspond to the integral quotient and the remainder of ${ NKM}_{obs}/{ NKM}_{monomer}$. Figure 6b shows an RKM-KMD plot of the PCL-1 sample. In this plot, the components with the same end-groups lined up the horizontal distribution can be condensed into specific (RKM, KMD) coordinate points. For example, PCL-diol, cyc-PCL, and carboxyl-PCL were condensed into (RKM, KMD) = (15, 0.024), (23, 0.024), and (41, 0.024), respectively. This plot makes it possible to visually recognize the distribution of different chemical structures (mainly end-group distribution) that result from specific synthetic processes.

**Figure 7:**
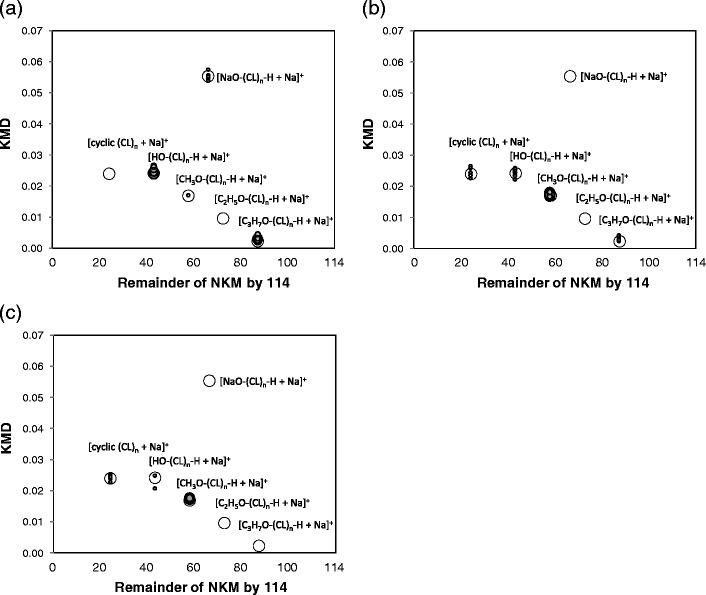
RKM-KMD plots of PCL-2 **(a)**, PCL-3 **(b)**, and PCL-4 **(c)**. Large blank circles are theoretical coordinate points of several possible PCL structures as indicated in the figures. The size of each dot indicates peak intensity. The peaks with more than 2% relative intensities were plotted

The RKM-KMD plot analysis was further applied to the profiling of several PCL samples synthesized through ring-opening polymerization of ε-caprolactone initiated with a variety of catalysts, as shown in Figure 7. The large blank circles on the plots indicate the theoretical points of possible structures such as α-carboxy, α-methoxy, α-ethoxy, and α-(iso)propioxy PCL and cyclic PCL. According to the supplier’s information, PCL-2 and PCL-3 were polymerized in the presence of aluminum isopropoxide as an initiator, whereas PCL-4 was done using triethyl ammonium. In the ring-opening polymerization in the presence of metal alkoxides as an initiator, the alcohol moiety would usually be introduced at the α-terminal of the PCL chains [25]. As expected, the plot of PCL-2 (Figure 7a) reveals PCL chains with an isopropoxide terminal (iPr-PCL) and carboxyl-PCL predominating. As by-products, small numbers of PCL chains with a methoxy terminal (Me-PCL) were also detected, whereas no cyc-PCL was observed. However, in spite of the use of the same catalyst, the main components of PCL-3 (Figure 7b) were PCL chains with a methyl terminal (Me-PCL) rather than iPr-PCL. The formation of cyc-PCL and carboxyl-PCL could be confirmed. These results suggest that the two PCL samples might be synthesized under different conditions. A possible reason for such differences might be the presence of alcohols as a co-initiator [26]. As for PCL-4 (Figure 7c), the supplier states that the main components are PCL chains with an ethoxy terminal (Et-PCL) because the ring-opening polymerization was performed in the presence of triethylalminum as a catalyst. However, the plot of Figure 7c makes it clear that the actual main components were Me-PCL together with cyc-PCL and carboxyl-PCL as minor components, with no Et-PCL detected. This sample might also have been polymerized in the presence of methanol as a co-initiator. As demonstrated above, the RKM-KMD plot revealed a variety of end-group combinations. Because end-group distributions tend to reflect the synthetic conditions of a given polymer, a database containing a set of theoretical RKM-KMD coordinates for possible polymer structures synthesized under certain conditions would be useful for quality control of the products.

## Conclusions

The results in this study demonstrated that MALDI spiral-TOFMS made it possible to perform KMD analysis for polymer characterization, which included discrimination of the polymer components in the blend, compositional analysis of copolymers, and profiling of the end-group distribution of polymers synthesized under different conditions. MALDI spiral-TOFMS, with its high resolving power and sufficient mass accuracy, opens the way to KMD analysis in the field of polymer characterization. One of the key advantages of this method is that the KMD plot visually represents patterns in the structural distribution of a given polymer without the need to perform peak assignment or peak picking. This feature is potentially useful for high-throughput profiling (or typing) of industrially-produced polymers, to inspect how the polymer was made and processed. Another possible utilization of KMD analysis for polymer characterization would be the investigation of polymer degradation processes, and an investigation along this line is now in progress.

## References

[1] Montaudo G, Samperi F, Montaudo MS (2006). Characterization of synthetic polymers by MALDI-MS. Prog. Polym. Sci..

[2] Gruendling T, Weidner S, Falkenhagen J, Barner-Kowollik C (2010). Mass spectrometry in polymer chemistry: A state-of-the-art up-date. Polym. Chem..

[3] Satoh T, Tsuno H, Iwanaga M, Kammei Y (2005). The design and characteristic features of a new time-of-flight mass spectrometer with a spiral ion trajectory. J. Am. Soc. Mass Spectrom..

[4] Satoh T, Sato T, Tamura J (2007). Development of a high-performance MALDI-TOF mass spectrometer utilizing a spiral ion trajectory. J. Am. Soc. Mass Spectrom..

[5] Tsujita T, Shintani T, Sato H (2013). alpha-Amylase inhibitory activity from nut seed skin polyphenols. 1. Purification and characterization of almond seed skin polyphenols. J. Agric. Food Chem..

[6] Tsujita T, Shintani T, Sato H (2014). Preparation and characterisation of peanut seed skin polyphenols. Food Chem..

[7] Sato H, Ishii Y, Momose H, Sato T, Teramoto K (2013). Structural characterization of free radical polymerized methacrylate ester copolymers using high-resolution MALDI-TOFMS with a spiral ion trajectory. Mass Spectrom.

[8] Kendrick E (1963). A mass scale based on CH_2_ = 14.0000 for high resolution mass spectrometry of organic compounds. Anal. Chem..

[9] Hughey CA, Hendrickson CL, Rodgers RP, Marshall AG, Qian KN (2001). Kendrick mass defect spectrum: A compact visual analysis for ultrahigh-resolution broadband mass spectra. Anal. Chem..

[10] Sleno L (2012). The use of mass defect in modern mass spectrometry. J. Mass Spectrom..

[11] Marshall AG, Rodgers RP (2004). Petroleomics: The next grand challenge for chemical analysis. Acc. Chem. Res..

[12] Stenson AC, Marshall AG, Cooper WT (2003). Exact masses and chemical formulas of individual Suwannee River fulvic acids from ultrahigh resolution electrospray ionization Fourier transform ion cyclotron resonance mass spectra. Anal. Chem..

[13] Sleighter RL, Hatcher PG (2007). The application of electrospray ionization coupled to ultrahigh resolution mass spectrometry for the molecular characterization of natural organic matter. J. Mass Spectrom..

[14] Lerno LA, German JB, Lebrilla CB (2010). Method for the identification of lipid classes based on referenced kendrick mass analysis. Anal. Chem..

[15] Lee H, An HJ, Lerno LA, German JB, Lebrilla CB (2011). Rapid profiling of bovine and human milk gangliosides by matrix-assisted laser desorption/ionization Fourier transform ion cyclotron resonance mass spectrometry. Int. J. Mass Spectrom..

[16] Riegner DE, Hofstadler SA, Laude DA (1991). Mass discrimination due to z-axis ion cloud coherence in the Fourier-transform mass-spectrometry trapped-ion cell. Anal. Chem..

[17] Dey M, Castoro JA, Wilkins CL (1995). Determination of molecular-weight distributions of polymers by MALDI-FTMS. Anal. Chem..

[18] Belov ME, Nikolaev EN, Harkewicz R, Masselon CD, Alving K, Smith RD (2001). Ion discrimination during ion accumulation in a quadrupole interface external to a Fourier transform ion cyclotron resonance mass spectrometer. Int. J. Mass Spectrom..

[19] Jaber AJ, Kaufman J, Liyanage R, Akhmetova E, Marney S, Wilkins CL (2005). Trapping of wide range mass-to-charge ions and dependence on matrix amount in internal source MALDI-FTMS. J. Am. Soc. Mass Spectrom..

[20] van Rooij GJ, Duursma MC, de Koster CG, Heeren RMA, Boon JJ, Schuyl PJW, van der Hage ERE (1998). Determination of block length distributions of poly(oxypropylene) and poly(oxyethylene) block copolymers by MALDI-FTICR mass spectrometry. Anal. Chem..

[21] Cox FJ, Qian KN, Patil AO, Johnston MV (2003). Microstructure and composition of ethylene-carbon monoxide copolymers by matrix-assisted laser desorption/ionization mass spectrometry. Macromolecules.

[22] Mize TH, Simonsick WJ, Amster IJ (2003). Characterization of polyesters by matrix-assisted laser desorption/ionization and Fourier transform mass spectrometry. Eur. J. Mass Spectrom..

[23] Jaber AJ, Wilkins CL (2005). Hydrocarbon polymer analysis by external MALDI Fourier transform and reflectron time of flight mass spectrometry. J. Am. Soc. Mass Spectrom..

[24] Sato H, Tao H, Ohtani H, Aoi K (2003). Characterization of poly(epsilon-caprolactone) by size exclusion chromatography/matrix-assisted laser desorption ionization-mass spectrometry (SEC/MALDI-MS). Kobunshi Ronbunshu.

[25] Dubois P, Ropson N, Jerome R, Teyssie P (1996). Macromolecular engineering of polylactones and polylactides. 19. Kinetics of ring-opening polymerization of epsilon-caprolactone initiated with functional aluminum alkoxides. Macromolecules.

[26] Mata-Mata JL, Baez JE, Gutierrez JA, Martinez-Richa A (2006). Ring-opening polymerization of lactones using RuCl2(PPh3)(3) as initiator: Effect of hydroxylic transfer agents. J. Appl. Polym. Sci..

